# Reptile Toll-like receptor 5 unveils adaptive evolution of bacterial flagellin recognition

**DOI:** 10.1038/srep19046

**Published:** 2016-01-07

**Authors:** Carlos G. P. Voogdt, Lieneke I. Bouwman, Marja J. L. Kik, Jaap A. Wagenaar, Jos P. M. van Putten

**Affiliations:** 1 Department of Infectious Diseases and Immunology, Faculty of Veterinary Medicine, Utrecht University, Yalelaan 1, 3584 CL, Utrecht, the Netherlands; 2 Department of Pathobiology, Faculty of Veterinary Medicine, Utrecht University, Yalelaan 1, 3584 CL, Utrecht, the Netherlands; 3 Central Veterinary Institute of Wageningen UR, Houtribweg 39, 8221 RA, Lelystad, the Netherlands

## Abstract

Toll-like receptors (TLR) are ancient innate immune receptors crucial for immune homeostasis and protection against infection. TLRs are present in mammals, birds, amphibians and fish but have not been functionally characterized in reptiles despite the central position of this animal class in vertebrate evolution. Here we report the cloning, characterization, and function of TLR5 of the reptile *Anolis carolinensis* (Green Anole lizard). The receptor (acTLR5) displays the typical TLR protein architecture with 22 extracellular leucine rich repeats flanked by a N- and C-terminal leucine rich repeat domain, a membrane-spanning region, and an intracellular TIR domain. The receptor is phylogenetically most similar to TLR5 of birds and most distant to fish TLR5. Transcript analysis revealed acTLR5 expression in multiple lizard tissues. Stimulation of acTLR5 with TLR ligands demonstrated unique responsiveness towards bacterial flagellin in both reptile and human cells. Comparison of acTLR5 and human TLR5 using purified flagellins revealed differential sensitivity to *Pseudomonas* but not *Salmonella* flagellin, indicating development of species-specific flagellin recognition during the divergent evolution of mammals and reptiles. Our discovery of reptile TLR5 fills the evolutionary gap regarding TLR conservation across vertebrates and provides novel insights in functional evolution of host-microbe interactions.

Toll-like receptors (TLRs) form a family of evolutionarily highly conserved innate immune receptors that play a crucial role in immune homeostasis and the response to infection^1^,^2^. TLRs are glycoproteins that typically consist of an extracellular sensor domain (ECD) composed of multiple leucine rich repeats (LRR), a transmembrane domain (TM) and an intracellular Toll/Interleukin-1 receptor (TIR) signalling domain^3^. The ECD senses the presence of conserved microbial structures in the environment and transduces this signal to the TIR domain which acts as a docking station for intracellular adapter proteins like Myeloid differentiation primary response gene 88 (MyD88). The formed complex then initiates a cascade of events that ultimately results in nuclear translocation of transcription factors like Nuclear factor kappa light chain enhancer of activated B cells (NF-κB) that direct the innate and adaptive immune response^4^.

Throughout evolution, selective pressures exerted by microbes have driven diversification of the TLR ECD, resulting in a family of distinct receptors that recognize a variety of mainly microbial ligands^5^. For example, TLR4 binds bacterial lipopolysaccharide^6^; TLR9 or 21 recognizes bacterial nucleic acid motifs^7^,^8^ and avian TLR15 is uniquely activated by microbial proteases via cleavage of the receptor ectodomain^9^. TLR5 senses flagellin subunits^10^ that make up the flagellum of certain bacterial species including *Salmonella enterica* and *Pseudomonas aeruginosa*. Besides structural diversity between TLR family members, coevolution with microbes has also led to adaptive evolution of individual TLRs^11^,^12^,^13^, leading to differential recognition of TLR ligands between animal species^14^,^15^,^16^.

Within the animal kingdom, the TLR repertoire varies among species. Regarding vertebrates, genome wide studies have identified 16 TLR types in lampreys compared to 20 in bony fish, 21 in amphibians and 10 in both humans and birds^4^,^17^,^18^,^19^,^20^. The dynamic evolution between and within TLR family members and the conservation of TLRs across highly diverse animals underlines the importance of TLRs throughout vertebrate evolution. However, one major gap in our knowledge on vertebrate TLR evolution is the complete lack of information about the structure, function, and ligand specificity of TLRs in any species of reptile. Reptiles have a unique physiology, being the only poikilothermic amniotes, and take a central position in vertebrate evolution^21^. The first reptiles evolved around 330 to 310 million years ago (Mya) from an amphibian-like ancestor^22^. Development of the amniotic egg and a water impermeable skin allowed these early reptiles to be the first vertebrates that could permanently colonize terrestrial habitats. This pioneering step must have brought the first reptiles into contact with the prehistorical terrestrial flora, fauna and microbiota that undoubtedly shaped the immune system of reptiles and descendant animals. Yet compared to other vertebrates our knowledge on the reptile immune system, especially concerning molecular insights in reptile microbe interactions, is marginal^21^.

In present study we report the molecular cloning, characterization and function of the first reptile TLR namely TLR5 of the ‘New world’ lizard *Anolis carolinensis* (acTLR5). Evidence is provided that acTLR5 is closely related to other TLR5 orthologs and responds to bacterial flagellin, even when expressed in human cells. Differential sensitivity of acTLR5 compared to human TLR5 to *Pseudomonas aeruginosa* but not *Salmonella* Enteritidis flagellins indicate host specific adaptation of flagellin recognition.

## Results

### Reptile cells respond to bacterial flagellin

**Figure 1:**
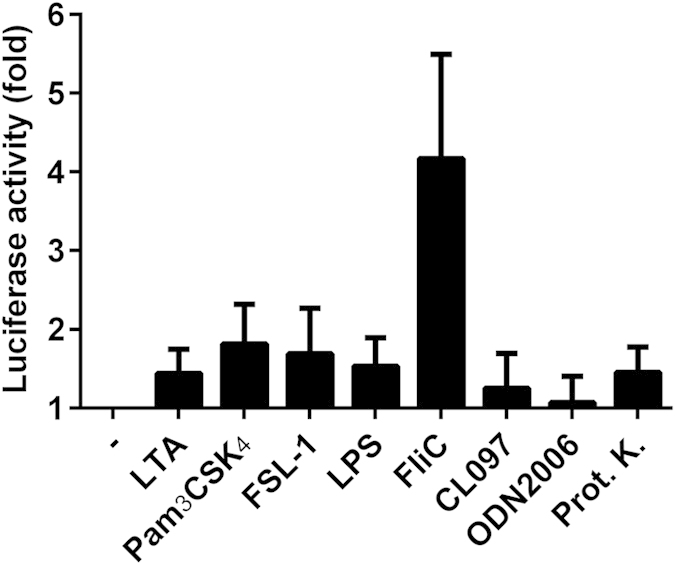
Flagellin stimulation activates NF-κB in IgH-2 cells. *Iguana iguana* IgH-2 cells were transfected with a NF-κB luciferase reporter plasmid and stimulated (5 h) with the following TLR ligands: LTA (1 μg ml^−1^), Pam_3_CSK_4_ (0.1 μg ml^−1^), FSL-1 (0.1 μg ml^−1^), LPS (0.1 μg ml^−1^), FliC (flagellin) (1 μg ml^−1^), CL097 (2 μg ml^−1^), ODN2006 (500 nM) and Proteinase K (2 ng ml^−1^). Data represent the fold increase of luciferase activity compared to the unstimulated control (−). Values are the mean ± s.e.m. of three independent experiments performed in duplicate.

To assess whether reptile cells respond to TLR ligands we first stimulated IgH-2 *Iguana iguana* cells carrying a NF-κB luciferase reporter plasmid with the canonical mammalian TLR ligands; LTA (TLR2), Pam_3_CSK_4_ (TLR2/1), FSL-1 (TLR2/6), LPS (TLR4), FliC (flagellin of *Salmonella enterica* serovar Enteritidis) (TLR5), CL097 (TLR7), ODN2006 (TLR9) and the avian TLR15 activator Proteinase K. None of these TLR agonists elicited significant NF-κB activity except for bacterial flagellin (Fig. 1). In search for the putative TLR receptor conferring this response, and by absence of the *I. iguana* whole genome sequence, we interrogated the whole genome sequence of the related model organism *Anolis carolinensis*^23^,^24^, using BLAST with mammalian and chicken TLR protein sequences as queries. This search yielded nine putative TLR orthologs including a putative TLR5 ortholog (Genbank accession number: XP003216083.1), which was designated as acTLR5.

### Expression and characterization of the ac*tlr5* gene

**Figure 2:**
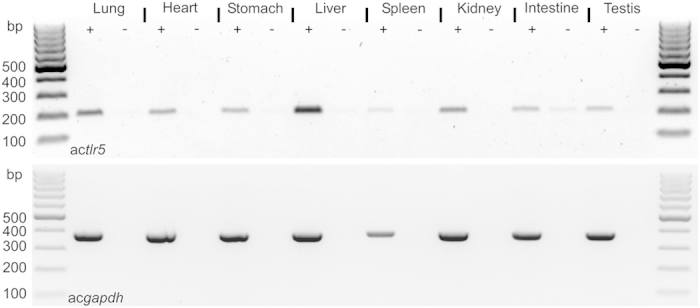
Expression of acTLR5 transcript in multiple tissues of *A. carolinensis*. RT-PCR analysis on total RNA extracted from the indicated tissues of an *A. carolinensis* lizard after reverse transcription into cDNA (+) or without the reverse transcription step (−). PCR amplified a 216 bp (base pair) fragment of ac*tlr5* or (as control) a 374 bp fragment of *A. carolinensis* glyceraldehyde 3-phosphate dehydrogenase (ac*gapdh*).

To verify that the putative acTLR5 ortholog is expressed *in vivo* in the Anolis lizard, we tested total mRNA isolated from different organs of an adult male for the presence of the ac*tlr5* transcript using RT-PCR with glyceraldehyde 3-phosphate dehydrogenase (ac*gapdh*) as a control. Transcripts of ac*tlr5* were detected in all the tissues tested including lung, heart, stomach, liver, spleen, kidneys, intestine and testis (Fig. 2), indicating that the gene product is expressed and may be functional in various tissues.

In order to examine the function of the acTLR5 we cloned the *tlr5* gene from genomic DNA of an adult male *A. carolinensis*. The gene consisted of a single exon encoding a protein of 871 amino acids that contained typical TLR domains. These included an ECD (residues 28 to 634) containing 24 LRRs (including N- and C-terminal LRR) as found in other TLR5 orthologs^25^, a TM domain (residues 647 to 665) and an intracellular TIR signalling domain (residues 697 to 840). The amino acid sequence differed from the *A. carolinensis* reference sequence at positions: 471 (H471L), 550 (V550A), 642 (S642P) and 658 (F658Y), suggesting the existence of polymorphisms in TLR5 of *A. carolinensis*.

Phylogenetic analysis using full-length protein sequences of different TLR types from several vertebrates including fish, amphibians, birds and mammals clustered acTLR5 with other TLR5 orthologs and in particular with chicken TLR5 (Supplementary Fig. S1). BLAST analysis with the ECD, TM and TIR domains as separate queries indicated that all three domains of acTLR5 were most similar to (predicted) TLR5 sequences of other reptiles and birds and least similar to fish TLR5 (Table 1), fully in line with the evolutionary relationships among these vertebrates.

### acTLR5 is functional in reptile but also in human cells

**Figure 3:**
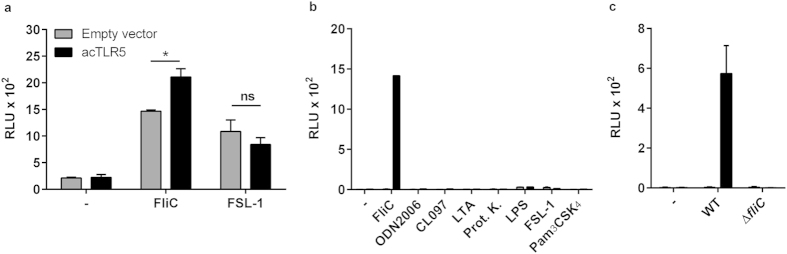
Response of acTLR5 expressed in reptile and human cells. (**a**) IgH-2 cells transfected with a NF-κB luciferase reporter plasmid and either empty vector or acTLR5 were stimulated with FliC (5 ng ml^−1^) or FSL-1 (250 ng ml^−1^) (10 h). *p < 0.05 by unpaired Students *t*-test. ns; not-significant. (**b**) NF-κB activation in human HeLa-57A cells transfected with empty vector or acTLR5 plasmid after 5 h of stimulation with the following TLR ligands: (−): unstimulated; FliC: 1 μg ml^−1^; ODN2006: 500 nM; CL097: 2 μg ml^−1^; LTA: 1 μg ml^−1^; Proteinase K: 2 ng ml^−1^; LPS: 0.1 μg ml^−1^; FSL-1: 0.1 μg ml^−1^; Pam_3_CSK_4_: 0.1 μg ml^−1^. (**c**) HeLa-57A cells transfected with empty vector or acTLR5 were incubated (5 h) with sterile LB medium (−), 2.5·10^4^ live wild-type *Salmonella* Enteritidis (WT) or the isogenic flagellin deficient strain (Δ*fliC*). Data represent the mean ± s.e.m. luciferase activity in relative light units (RLU) of three independent experiments performed in duplicate (**a,c**) or the mean RLU of a representative of three independent experiments performed in duplicate (**b**).

Evidence for the function of acTLR5 was sought by introducing an expression vector carrying ac*tlr5* (or a control plasmid without insert) together with a NF-κB luciferase reporter plasmid into reptile IgH-2 cells. Stimulation of the mock-transfected cells with *S.* Enteritidis flagellin (FliC) increased NF-κB activity in these cells, confirming the results depicted in Fig. 1. However, stimulation with *S.* Enteritidis flagellin significantly increased NF-κB activity in acTLR5 transfected cells (*p* < 0.05) (Fig. 3a), indicating that recombinant acTLR5 is functional in the transfected reptile cells and responds to flagellin. To ensure the specificity of this response, cells were stimulated with FSL-1, a synthetic lipoprotein known to be recognized by TLR2 and TLR6 heterodimers. A high dose of FSL-1 yielded similar responses in empty vector and acTLR5 transfected cells (Fig. 3a) confirming the specificity of the flagellin-induced acTLR5 response.

Reptiles and mammals have evolved independently over more than 300 million years^22^. Yet, a sequence alignment of acTLR5 with human and other vertebrate TLR5 orthologs indicated strong conservation across vertebrates of a critical proline^15^ and tyrosine^26^ residue as well as a phosphorylation motif^27^ in the TLR5 signalling domain (Supplementary Fig. S2). To determine whether TLR5 signalling has evolved under strong functional constraint, the functioning of acTLR5 was determined in human HeLa-57A cells which do not endogenously express TLRs and stably express the NF-κB luciferase reporter^28^. Stimulation with FliC, and not with other TLR ligands, yielded a strong increase in NF-κB activity in acTLR5 transfected human cells compared to control cells carrying empty vector (Fig. 3b). This functionality of reptile TLR5 in human cells strongly suggests that the expression and trafficking of the receptor and its signalling properties as well as its ligand specificity have been functionally conserved across the reptile and mammalian lineage.

Finally, to verify that acTLR5 was also able to recognize native (non-recombinant) flagellin we incubated acTLR5 transfected cells with live wild-type *S.* Enteritidis (WT) or its isogenic flagellin deficient derivative (Δ*fliC*). Only incubation with wild-type *S.* Enteritidis resulted in NF-κB activation in an acTLR5 dependent manner, confirming that TLR5 is a bonafide reptile receptor for bacterial flagellin (Fig. 3c).

### Reptile and human TLR5 recognize the D1 domain in flagellin

Now that we had identified acTLR5 as a specific receptor for bacterial flagellin, we examined the conservation of residues involved in flagellin binding by aligning acTLR5 (and also chicken, African clawed frog and human TLR5) with zebrafish TLR5b of which the crystal structure in complex with flagellin has been determined^29^. The alignment showed that only 40% (18/45) of the zebrafish TLR5b-flagellin interacting residues resemble the residues at the same positions in acTLR5 and the other vertebrate TLR5 sequences (Supplementary Fig. S2), suggesting a differential basis for the structural recognition of flagellin among these vertebrates.

**Figure 4:**
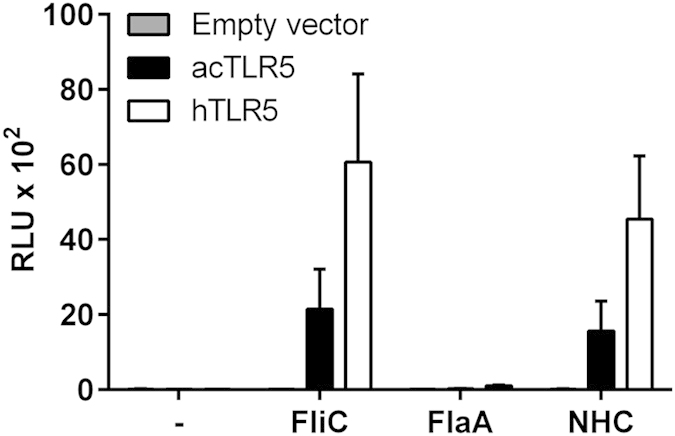
Activation of acTLR5 by the D1 domain of *Salmonella* FliC. HeLa-57A cells transfected with empty vector, acTLR5 or human TLR5 (hTLR5) were stimulated (5 h) with *S.* Enteritidis FliC, *C. jejuni* FlaA or chimeric NHC flagellin (1 μg ml^−1^). Results represent the mean ± s.e.m. luciferase activity as relative light units (RLU) of three independent experiments performed in duplicate.

To determine whether the structural differences in TLR5 have influenced flagellin recognition throughout the divergent evolution of reptiles and mammals we mapped the domain of flagellin that is recognized by acTLR5. For this, we took advantage of the fact that the D1 domain of flagellins of *β-* and *γ-* Proteobacteria (incl. *Salmonella*, and *Pseudomonas* species) activate TLR5, whereas, due to compositional changes, the D1 domain of flagellins of *α-* and *ε-* Proteobacteria (incl. *Campylobacter* species) escapes recognition by TLR5^30^,^31^. acTLR5 or human TLR5 (hTLR5) were transfected in HeLa-57A cells and stimulated with purified recombinant *S.* Enteritidis flagellin (FliC) or *Campylobacter jejuni* flagellin (FlaA). This showed that *S.* Enteritidis FliC but not *Campylobacter* FlaA activated NF-κB in both acTLR5 and hTLR5 transfected cells (Fig. 4). To ascertain that the unresponsiveness of acTLR5 and hTLR5 to *Campylobacter* FlaA involved the FlaA D1 domain, we stimulated both TLRs with NHC flagellin. NHC is a chimeric flagellin based on *Campylobacter* FlaA in which the D1 domain was exchanged for the *S.* Enteritidis FliC D1 domain^31^. Indeed, this swapping of the D1 domain restored the activation of hTLR5 and acTLR5 (Fig. 4), indicating that both receptors recognize the D1 region of *Salmonella* but not *Campylobacter* flagellin (Fig. 4) and thus that this ability is conserved between reptiles and humans. The inability of acTLR5 and hTLR5 to recognize *Campylobacter* flagellin may further indicate that evasion of TLR5 detection by *Campylobacter* developed before the divergence of reptiles and mammals.

### Lysate of *Pseudomonas* activates reptile but not human TLR5

**Figure 5:**
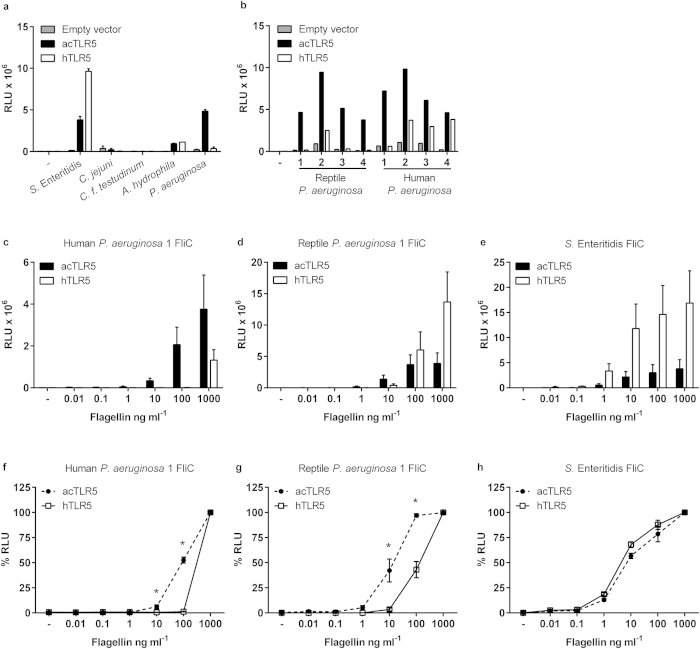
Differential recognition of *Pseudomonas* flagellins by acTLR5 and hTLR5. (**a**) HeLa-57A cells transfected with acTLR5, hTLR5 or empty vector were stimulated (5 h) with lysate (1 μg ml^−1^ total protein) of the indicated bacterial species, or (**b**) four different reptile and four different human *P. aeruginosa* isolates. **(c,d,e)** acTLR5 or hTLR5 transfected cells were stimulated (5 h) with the indicated concentrations of purified his-tagged flagellin of human *P. aeruginosa* isolate 1 (**c**) or reptile *P. aeruginosa* isolate 1 (**d**) or *S.* Enteritidis (**e**). (**f–h**) Relative sensitivity plots calculated from figures **(**c,d,e**)** showing the % RLU of acTLR5 and hTLR5 for each of the indicated concentrations of flagellin of human *P. aeruginosa* isolate 1 (**f**), reptile *P. aeruginosa* isolate 1 (**g**) or *S.* Enteritidis (**h**). The response to 1000 ng ml^−1^ flagellin was set at 100%. Values show the mean ± s.e.m. luciferase activity as relative light units (RLU) of two independent experiments (**a**), a representative of two independent experiments (**b**) or mean ± s.e.m. of three independent experiments (**c–h**) all performed in duplicate. *p < 0.05 by unpaired Students *t*-test.

Despite recognition of the same flagellin D1 domain, chicken, mouse and human TLR5 respond differently to flagellins of various bacterial species^14^,^15^, suggesting host specific adaptations in bacterial flagellin recognition. To determine whether specific adaptations in flagellin recognition have also occurred in reptiles, we compared the response of acTLR5 transfected HeLa-57A cells with hTLR5 transfected cells to stimulation with bacterial lysate of *S.* Enteritidis, *C. jejuni* and three motile reptile isolates i.e., a *Campylobacter fetus* subsp*. testudinum*^32^, *Aeromonas hydrophila* and *Pseudomonas aeruginosa.* For *S.* Enteritidis and *C. jejuni* the use of lysate closely resembled the response to purified flagellins (compare Fig. 5a to Fig. 4). Stimulation of the cells with the reptile *C. fetus* subsp*. testudinum* lysate did not activate acTLR5 or hTLR5 (Fig. 5a) suggesting a similar evasion of TLR5 recognition by this reptile strain as noted for mammalian and chicken derived *Campylobacter* strains^31^. The lysate of *A. hydrophila* activated acTLR5 and hTLR5 equally well (Fig. 5a). However in clear contrast, reptile derived *P. aeruginosa* (isolate 1) potently activated acTLR5 but failed to activate hTLR5 (Fig. 5a). Additional analysis using three extra reptile (isolates 2–4) and also four human *P. aeruginosa* isolates (isolates 1–4) indicated stronger activation of acTLR5 than hTLR5 by *P. aeruginosa* isolates, regardless of their reptile or human origin (Fig. 5b). The opposite response of these TLRs to the *Pseudomonas* and *Salmonella* lysates indicates that differential recognition of the lysates was not due to variable receptor expression.

### acTLR5 is more sensitive than hTLR5 to *Pseudomonas* flagellin

To verify that flagellin was the key determinant in the differential recognition of *P. aeruginosa* lysates by acTLR5 and hTLR5 and to exclude the destructive effect of flagellin degrading proteases potentially present in the lysates^33^, we cloned and purified recombinant flagellin of reptile and human *P. aeruginosa* isolate 1. Stimulation of transfected HeLa-57A cells with low concentrations (0.1–10 ng ml^−1^) of these purified *P. aeruginosa* flagellins revealed again stronger activation of acTLR5 compared to hTLR5 (Fig. 5c,d) and an opposite effect for *S.* Enteritidis flagellin (Fig. 5e). At high concentrations (100–1,000 ng ml^−1^), reptile but not human *P. aeruginosa* flagellin did yield a potent hTLR5 response.

The differential dose-dependent responses by acTLR5 and hTLR5 suggested that the receptors recognize the purified flagellins with a different sensitivity. To substantiate the apparent different sensitivity of acTLR5 and hTLR5 to the purified flagellins we set the response to 1,000 ng ml^−1^ flagellin at 100%. This revealed that the receptors had a similar relative sensitivity to *S.* Enteritidis flagellin (Fig. 5h). However, compared to hTLR5, acTLR5 showed a higher relative sensitivity to the human *P. aeruginosa* flagellin (*p* < 0.05) (Fig. 5f). Higher relative sensitivity of acTLR5 was also noted for the reptile *P. aeruginosa* flagellin (*p* < 0.05) (Fig. 5g), despite the fact that high doses of this flagellin induced stronger activation of hTLR5. Overall, these results show that acTLR5 is more sensitive than hTLR5 to *P. aeruginosa* but not *S.* Enteritidis flagellin.

## Discussion

Reptiles form a large group of vertebrates with a central position in vertebrate evolution and a unique physiology, being the only ectothermic amniotes. Despite this, relatively few studies have investigated the reptile immune system and detailed molecular characterizations of reptile immune molecules are scarce. Here we report a detailed functional characterization of the first TLR in reptiles. Our characterization of TLR5 of the lizard *A. carolinensis* fills the evolutionary gap of functional TLRs across vertebrates and provides a novel view on the reptile immune system at a molecular level. Evidence is provided that acTLR5 is expressed and functional in reptile as well as human cells and responds to bacterial flagellin. Our results indicate that TLR5 structure, function and signalling are highly conserved throughout evolution, although differences in relative sensitivity of reptile and human TLR5 to *Pseudomonas* but not *Salmonella* flagellin point to bacterial species dependent adaptations in flagellin recognition by reptile and human TLR5.

The reptile *tlr5* gene was cloned from an *Anolis carolinensis* lizard. Support for its identification as *tlr5* ortholog included a strong phylogenetic relationship of the full-length protein with the well-characterized chicken TLR5^15^,^31^. The ECD and TIR domain of the cloned acTLR5 were highly similar to a putative TLR of the Burmese python (snake), suggesting that the gene is present in other reptiles as well. Lizards, snakes and tuatara form the group of Lepidosauria that diverged approximately 270 Mya from their bird and crocodile sister group; the Archosauria^34^. Lizards and snakes thereafter diverged approximately 180 Mya^35^,^36^. The phylogeny of these species is reflected by the high similarity of acTLR5 with the putative snake and chicken TLR5, suggesting that TLR5 underwent a constrained evolution according to species divergence.

Functional evidence for identifying the cloned Anolis gene as a TLR5 ortholog was provided by the responsiveness of acTLR5 transfected cells to bacterial flagellin, thus far the only known TLR5 ligand. Activation of NF-κB in acTLR5 expressing cells was observed upon stimulation with wild type but not flagellin-deficient *Salmonella* as well as with purified recombinant *Salmonella* and *Pseudomonas* flagellins, thereby excluding non-specific activation of NF-κB. The results indicate that acTLR5 senses flagellins of different bacterial species and is capable of initiating a signalling cascade required to evoke an immune response. In mammals, flagellin recognition by TLR5 is indispensable for an adequate immune response to infection with flagellated bacteria^37^,^38^,^39^,^40^. As *A. carolinensis* tissues express the ac*tlr5* gene *in vivo* (Fig. 2) acTLR5 may have a similar function in reptiles.

A striking finding that underpins the evolutionary conservation of the TLR system is the functional expression of reptile TLR5 in a human cell background. The first step in TLR5 mediated NF-κB activation is the recruitment of the intracellular MyD88 adapter protein to the TLR5 TIR domain^41^. Comparison of the TIR domains of reptile and human TLR5 revealed a high overall sequence similarity (85%) and conservation of specific amino acid residues that are critical for TLR5 signalling^15^,^26^,^27^. In addition, both the TIR domain and MyD88 have been shown to evolve under strong functional constraint^42^,^43^,^44^. Together, this may explain the successful activation of NF-κB by acTLR5 in human cells. The compatibility of reptile TLR5 with human intracellular proteins suggests that the TLR5 signalling system was already functional in the common ancestor of reptiles and mammals and provides support for the functionally constrained evolution of TLR5 signalling at least throughout the divergent evolution of reptiles and mammals. Here it may be noteworthy that efforts to functionally express intact TLR5 from fish or amphibians in human cells have thus far not been reported.

Bioinformatics analysis indicated that the ECD of acTLR5 contained a N- and C-terminal LRR separated by 22 consecutive LRRs which is a typical feature of chicken^15^ and other vertebrate TLR5 orthologs^25^. In line with the apparent conserved structure of the ECD, both reptile and human TLR5 recognized and responded to the D1 domain of *Salmonella* but not *Campylobacter* flagellin. This finding demonstrates that throughout 300 million years of divergent evolution, reptile and human TLR5 have conserved the ability to recognize flagellin at its D1 domain and hence the flagellin D1 domain of certain bacterial species has remained a critical activator of TLR5.

Interestingly, despite different amino acid compositions of the ECD, reptile and human TLR5 showed equal sensitivity to flagellin of *Salmonella enterica* serovar Enteritidis. Pet reptiles are frequently reported as carriers of zoonotic *Salmonella* serovars that can cause salmonellosis in humans but are generally considered non-pathogenic in healthy reptiles^45^,^46^,^47^,^48^,^49^. The principles underlying resistance or tolerance of reptiles to *Salmonella* are unknown but may relate to the poikilothermic nature of reptiles since *Salmonella* virulence is influenced by environmental temperature^50^,^51^. Yet, the fact that reptile and human TLR5 show a similar relative sensitivity to *S.* Enteritidis flagellin may suggest that flagellin recognition does not play a significant role in the differential susceptibility to *Salmonella* infection observed between reptiles and humans.

In contrast to *Salmonella* flagellin, reptile and human TLR5 showed a differential sensitivity to flagellin of *P. aeruginosa* clinical isolates. *P. aeruginosa* is a common bacterium that resides in diverse environments including water and soil and is an opportunistic pathogen of both reptiles and humans^52^,^53^. Why reptile TLR5 is more sensitive to *P. aeruginosa* flagellin than human TLR5 remains to be elucidated but it may suggest that throughout host-microbe coevolution, *P. aeruginosa* has exerted a stronger selective pressure on the evolution of acTLR5 than on hTLR5. Indeed, *in silico* studies indicate that among primates^54^ and galloanserae birds^13^ TLR5 undergoes diversifying, adaptive evolution through positive selection, a process most likely driven by host specific coevolution with flagellated bacteria. A similar process in reptiles may explain the observed differences in *P. aeruginosa* flagellin recognition between the Anolis and human TLR5.

## Methods and Materials

### Isolation of *Anolis carolinensis* DNA and RNA

Anolis tissue samples were obtained from a healthy male *Anolis carolinensis* lizard that had been euthanized by intra-coelomic injection of pentobarbital (200 mg kg^−1^ BW, Euthanimal^®^, Alfasan International, The Netherlands). Organs were directly frozen in liquid nitrogen. Genomic DNA was isolated using the high pure template kit (Roche) according to the manufacturer’s instructions. RNA was extracted from tissue lysed with RLT buffer (1% *β*-mercaptoethanol) (Qiagen) in 1.4 mm Fastprep lysing matrix tubes (MPbio) in a Magna Lyser centrifuge (6,500 × *g*, 40 s, RT) (Roche). Total RNA was isolated using the RNeasy mini kit (Qiagen) following the manufacturer’s instructions, treated with DNase I (1 U mg^−1^ RNA, Thermo Scientific) and stored at −80 °C until use.

### Ethics statement

Euthanasia of the Anolis lizard was performed by a veterinarian specialized in reptiles (M.K, Diplomat European College Zoological Medicine, herpetology) and was in accordance with the guidelines in the Directive 2010/63/EU of the European Parliament and of the September 2010 Council on the protection of animals used for scientific purposes (http://eur-lex.europa.eu/legal-content/EN/TXT/?uri=CELEX:32010L0063). The procedure was approved by the Animal Ethics Committee of Utrecht University (study number 2014.II.04.031).

### Cloning of *A.* *carolinensis tlr5*

The *A. carolinensis tlr5* gene (ac*tlr5*) was amplified from genomic DNA (500 ng) by PCR in 50 μl volume containing 1X Phusion polymerase buffer, dNTP’s (0.2 mM each), MgCl_2_ (50 mM), Phusion hot start II high fidelity polymerase (1 Unit, Thermo Scientific) and 20 μM of forward (5′-CCGGATCCATGAAAAAGATGCTTCATTATCTCTTC-3′) and reverse (5′-CCGCGGCC**GC**AAGAGATTGTGACTACTTT-3′) primer (Life Technologies). Underlined sequences in the forward and reverse primer indicate BamHI and NotI restriction sites, respectively. The bold GC in the reverse primer substituted an AG in the *tlr5* gene, thereby replacing the terminal stopcodon for a cysteine. PCR conditions were: one cycle for 1 min at 98 °C followed by 35 cycles of 30 s at 98 °C, 30 s at 54 °C, 90 s at 72 °C and one final extension step of 10 min at 72 °C. The PCR product was purified from gel using the GeneJet gel extraction kit (Thermo Scientific) and ligated into a pTracer-CMV2ΔGFP/3×FLAG^8^ using the BamHI and NotI restriction sites, yielding pTracer 3 × FLAG-ac*tlr5* carrying ac*tlr5* with a C-terminal 3×FLAG tag. The plasmid was propagated in DH5-*α*. The cloned ac*tlr5* gene sequence was verified by DNA sequencing (Macrogen). The sequence was deposited in Genbank (accession number: KT347095).

### Reverse transcriptase PCR on ac*tlr5* mRNA from various tissues

First strand cDNA was created of 1 μg total RNA with oligo (dT)_18_ primers using the RevertAid First Strand cDNA Synthesis kit (Thermo Scientific). Non-reverse transcribed RNA served as control. PCR on cDNA and control samples was performed in 50 μl volume containing 1X DreamTaq polymerase buffer, dNTP’s (0.2 mM each), DreamTaq Green DNA polymerase (1.25 Units, Thermo Scientific), template cDNA (10 ng) and 20 μM of ac*tlr5* forward (5′-GCATGAATTCCTTGGGCACTCTG-3′) and reverse (5′-GGGCCACATCCCAACCATTAC-3′) primer or *A. carolinensis* GAPDH (ac*gapdh*) forward (5′-GAGAGGAGCTTCTCAGAACATC-3′) and reverse (5′-GACAATGCGGTTGCTGTATC-3′) primer. PCR conditions were: one cycle of 3 min at 95 °C followed by 35 cycles of 30 s at 95 °C, 30 s at 55 °C, 1 min at 72 °C and followed by one final extension step of 10 min at 72 °C. PCR products were analysed using 2% agarose gels.

### acTLR5 bioinformatics analysis

Amino acid sequence comparison was performed using NCBI-BLAST (http://blast.ncbi.nlm.nih.gov/Blast.cgi). Multiple sequence alignment was conducted using Clustal W^55^ (http://www.ebi.ac.uk/Tools/msa/clustalw2/) with default settings. MEGA6 software^56^ was used to construct the phylogenetic tree. Leucine rich repeats in the predicted acTLR5 protein sequence were identified by manual sequence analysis as described by Matsushima *et al.*^25^ and by use of the Leucine Rich Repeat Finder database (http://www.lrrfinder.com/). Transmembrane domains were predicted with the TMpred server (http://embnet.vital-it.ch/software/TMPREDform.html). A TIR domain was predicted by identifying five alternating *β*-sheets and *α*-helices^57^ using the Proteus Protein Structure Prediction server (http://wks80920.ccis.ualberta.ca/proteus/).

### Bacterial strains

The following bacterial strains were grown (37 °C, 18 h) on Luria-Bertani (LB) agar plates or in 5 ml of LB broth (Biotrading) at 160 rpm: *Escherichia coli* DH5-*α*, *E. coli* BL21 star (DE3), *Salmonella enterica* serovar Enteritidis (referred to as *S.* Enteritidis) strain 90-13-706 (CVI, Lelystad, The Netherlands), *S.* Enteritidis 90-13-706 isogenic *fliC* mutant^58^, *Aeromonas hydrophila* (turtle isolate, Utrecht University), four *Pseudomonas aeruginosa* from reptiles (one lizard, three snake isolates, Utrecht University), and four human *P. aeruginosa* isolates (University Medical Center Utrecht). *Campylobacter jejuni* strain 81116 (NCTC: 11828), and *Campylobacter fetus* subsp. *testudinum* (reptile isolate^32^) were grown (37 °C, 18 h) under micro-aerobic conditions (80% N_2,_ 7.5% H_2,_ 7.5% CO_2,_ 5% O_2_) on saponin agar plates or in LB broth at 160 rpm.

### Preparation of bacterial cell lysates

Single colonies of the bacterial species described above were grown (37 °C, 20 h) in 5 ml LB broth at 160 rpm and placed on ice. After microscopic confirmation of motility all cultures were normalized to an OD_550_ of 2, pelleted by centrifugation (5,000 × *g*, 30 min, 4 °C), washed with 1 ml Dulbecco’s phosphate buffered saline (DPBS, Sigma), briefly vortexed, and collected by centrifugation (5,000 × *g*, 30 min, 4 °C). Pellets were re-dissolved in 2 ml DPBS and placed for 1 h at 70 °C. Heat killed bacteria were sonicated (6 × 15 s, Vibra-cell, Sonics, USA) and centrifuged (14,000 × *g*, 40 min, 4 °C). Lysate supernatants were stored at −20 °C until use. Protein concentration of lysates was determined by BCA assay (Thermo Scientific).

### Construction, expression and purification of recombinant His-tagged flagellins

Construction of recombinant His-tagged flagellin of *S.* Enteritidis (FliC), *C. jejuni* (FlaA) and chimeric NHC has been described previously^15^,^31^. The flagellin gene of both reptile and human *P. aeruginosa* isolate 1 was amplified from genomic DNA by PCR in 50 μl volume containing 1X Dreamtaq polymerase buffer, dNTP’s (0.2 mM each), Dreamtaq polymerase (1 Unit) and 20 μM of forward (5′-AAACCATGGCCTTGACCGTCAACAC-3′) and reverse (5′-AAAGAGCTCGCGCAGCAGGCTCAGAAC-3′) primer. Underlined sequences in the forward and reverse primer indicate NcoI and SacI restriction sites, respectively. PCR conditions were: one cycle for 3 min at 95 °C followed by 35 cycles of 30 s at 95 °C, 30 s at 64 °C, 2 min at 72 °C and a final extension step for 10 min at 72 °C. PCR products were ligated into the pET101/D-TOPO (Promega) expression vector using NcoI and SacI restriction enzymes. Ligation into the pET101/D-TOPO vector added a C-terminal His-tag to the flagellin gene and the plasmids were transformed into *E. coli* BL21 star (DE3).

Protein expression was induced by growing log phase cultures in the presence of 1 mM IPTG (Thermo Scientific) for 4 h at 37 °C. For flagellin purification bacteria were pelleted (4,400 × *g*, 15 min, 4 °C), resuspended in 10 ml cold DPBS with protease inhibitor cocktail (Roche), spun down (4,400 × *g*, 15 min, 4 °C) and incubated (RT) under end-over-end rotation for 16 h in 8 M urea buffer (8 M urea, 100 mM NaH_2_PO_4_, 100 mM Tris-HCl, pH 8). After removal of cell debris (5,300 × *g*, 30 min, RT) supernatant was incubated with Ni^2 + ^-NTA agarose beads (Qiagen). After 2 h the beads were washed with 4 × 4 ml of 8 M urea buffer pH 6.3. Flagellins were eluted with 4 × 0.5 ml of 8 M urea buffer pH 5.9 followed by 4 × 0.5 ml of 8 M urea buffer pH 4.5. Collected fractions were checked for purity on SDS-PAGE and pure fractions were pooled and concentrated using Amicon YM-30 filters (Millipore). Protein concentration was measured by BCA assay. Concentrated flagellins were diluted to the desired concentration and stored (−20 °C) as aliquots in 4 M urea, 100 mM NaH_2_PO_4_, 10 mM Tris-HCl, pH 9.

### Cell culture

HeLa-57A cells that are stably transfected with a NF-κB luciferase reporter construct^59^ were routinely propagated in Dulbecco’s modified eagle medium (DMEM) plus 5% fetal calf serum (FCS, Bodinco) at 37 °C and 10% CO_2_. Green iguana (*Iguana iguana*) heart cells (IgH-2, ATCC: CCL-108) were grown in minimal essential medium with Hank’s salts (MEM) and 10% FCS at 30 °C in air. Cells were passaged twice a week.

### Transient transfection of cells

HeLa-57A cells and IgH-2 cells were grown to ± 80% confluence in a 6-well plate and transfected using Fugene HD transfection reagent (Promega) according to the manufacturer’s instructions. HeLa-57A cells and IgH-2 cells were transfected with 1 μg of pTracer 3xFLAG-ac*tlr5* or human (h)*tlr5*^15^ at a lipid to DNA ratio of 3:1 (HeLa-57A) or 4:1 (IgH-2). Empty pTracer 3xFLAG was used for mock transfections. IgH-2 were additionally transfected with 1 μg of NF-κB luciferase reporter plasmid.

### Luciferase assay

Twenty-four hours after transfection cells were re-distributed in a 48-well plate. After 24 h cells were washed twice with medium without FCS and stimulated with the indicated TLR ligands or live bacteria in 500 μl medium without FCS (for stimulation with LPS, medium did contain FCS). After 5 h at 37 °C (HeLa-57A) or 10 h at 30 °C (IgH-2), cells were washed with DPBS and lysed with reporter lysis buffer (100 μl, Promega) at −80 °C for at least 1 h. After thawing, cell lysate (20 μl) was mixed with luciferase reagent (50 μl, Promega) and luciferase activity was measured in a luminometer (TD20/20, Turner designs). Experiments with bacterial lysates and purified *P. aeruginosa* flagellins were performed in 96-well plates in 250 μl volumes. Cells were lysed in 50 μl reporter lysis buffer. Luciferase activity in these experiments was measured with a TriStar^2^ luminometer (Berthold) by mixing 15 μl cell lysate with 37 μl luciferase reagent. Values obtained from the TriStar^2^ were 1000 times higher compared to the values obtained from the TD20/20 but relative sensitivity and accuracy between the two luminometers was equal. Results were expressed in relative light units (RLU) or % RLU in experiments with purified *P. aeruginosa* flagellins. Percent RLU was calculated by dividing the RLU obtained from each concentration of flagellin over the value obtained from stimulation with 1 μg ml^−1^ flagellin which was set at 100%.

### Statistics

Statistical analysis were performed using Graphpad 6 (Prism) software. Differences between two groups were tested with unpaired Student *t*-tests. A probability (*p*) value of < 0.05 was considered significant.

## Additional Information

**Accession codes:** The *Anolis carolinensis* TLR5 sequence was deposited in GenBank under accession code: KT347095.

**How to cite this article**: Voogdt, C. G.P. *et al.* Reptile Toll-like receptor 5 unveils adaptive evolution of bacterial flagellin recognition. *Sci. Rep.* **6**, 19046; doi: 10.1038/srep19046 (2016).

## Supplementary Material

Supplementary figures S1 and S2

## Figures and Tables

**Table 1:** Similarity (%) of acTLR5 domains with several vertebrate TLR5 orthologs.

TLR5 Species	Accession number	ECD^b^	TM^c^	TIR^d^
*Python bivittatus* (r)^a^	XP_007434471.1	76	68	95
*Chelonia mydas* (r)	EMP25733.1	74	73	94
*Alligator mississippiensis* (r)	XP_006270945.1	72	77	93
*Gallus gallus* (b)	ABW07794.1	70	64	90
*Columba livia* (b)	AIK67343.1	70	77	92
*Anser anser* (b)	AFP65787.1	68	72	91
*Xenopus leavis* (a)	NP_001088449.1	65	47	90
*Bos taurus* (m)	ABC68311.1	65	69	85
*Homo sapiens* (m)	NP_003259.2	64	69	85
*Mus musculus* (m)	AAI25262.1	63	69	84
*Oncorhynchus mykiss* (f)	NP_001118216.1	58	53	80
*Takifugu rubripes* (f)	AAW69374.1	57	54	77
*Danio rerio* (f)	NP_001124067.1	56	57	78

^a^r: reptile; b: bird; a: amphibian; m: mammal; f: fish.

^b^ECD: extracellular domain, residues 28 to 634.

^c^TM: transmembrane domain, residues 647 to 665.

^d^TIR: Toll/interleukin-1 receptor domain, residues 697 to 840.
