# Supplementary material for: Reptile Toll-like receptor 5 unveils adaptive evolution of bacterial flagellin recognition
Source: Sci Rep. 2016 Jan 7;6:19046. doi: 10.1038/srep19046 (PMC4703953; doi:10.1038/srep19046)
Supplement: Supplementary figures S1 and S2 [file srep19046-s1.pdf]

Reptile Toll-like receptor 5 unveils adaptive evolution of bacterial flagellin recognition

Carlos G.P. Voogdt, Lieneke I. Bouwman, Marja J.L. Kik, Jaap A. Wagenaar, Jos P.M. van Putten

Supplementary figure S1.

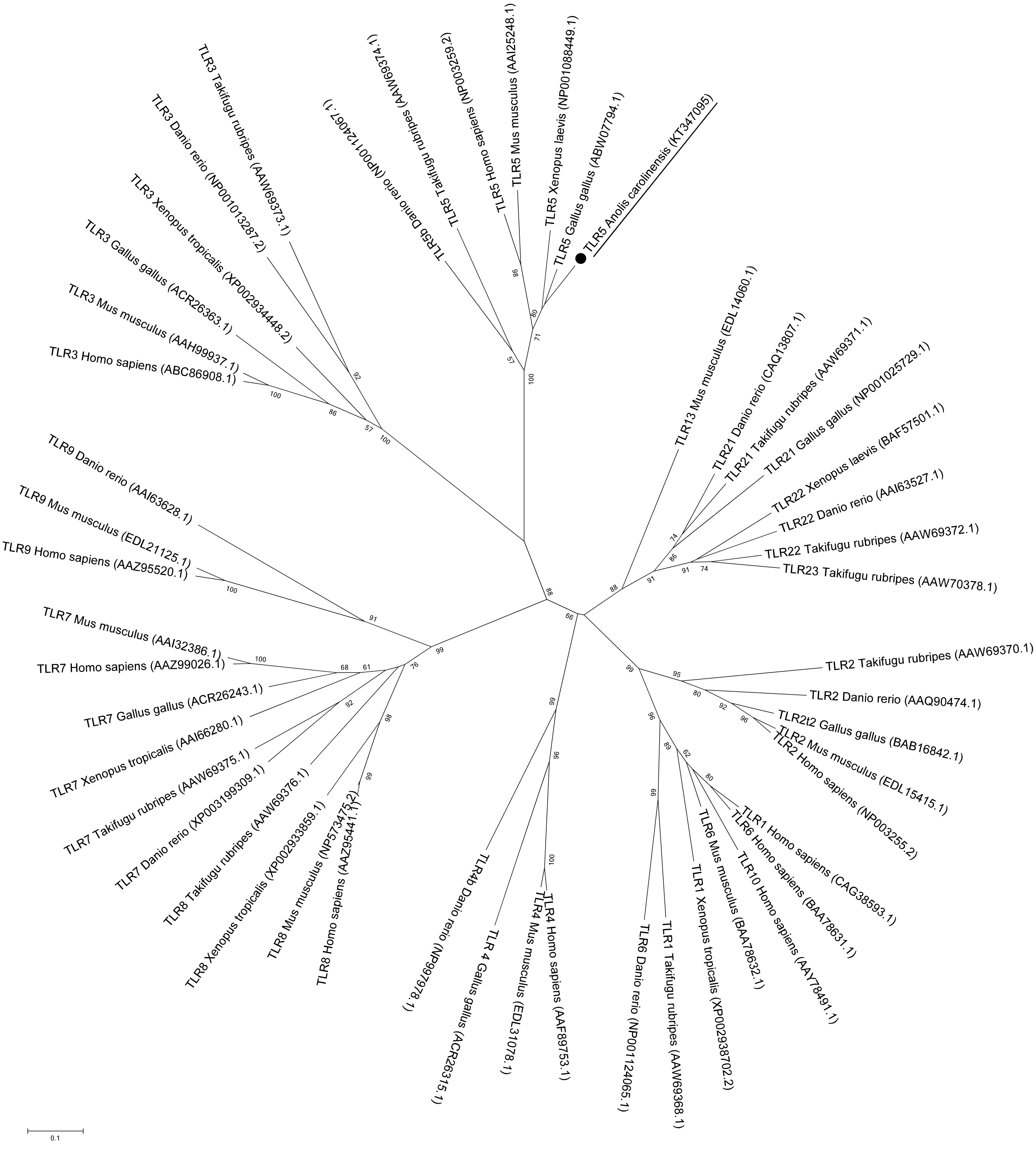

Supplementary figure S1. Phylogenetic position of *A. carolinensis* TLR5.

MEGA6 software[1] was used to construct a phylogenetic tree of different TLR families according to the Neighbour-Joining method with a bootstrap analysis of 10,000 iterations to indicate the relative support for each branch. acTLR5 is underlined and indicated with ●. GenBank accession numbers of the TLR protein sequences are given in brackets following the species name. Only bootstrap values of > 50 are shown. The tree is drawn to scale with the scale bar representing the evolutionary distance of 0.1 amino acid substitutions per site in the TLRs.

1. Tamura, K., Stecher, G., Peterson, D., Filipski, A. & Kumar, S. MEGA6: Molecular Evolutionary Genetics Analysis Version 6.0. *Mol Biol Evol* **30,** 2725–2729 (2013).

Supplementary figure S2.

```
                          Signal peptide           LRRNT
acTLR5    -----------MKKMLHYLFIFLIGMRHACREILAIPLCSVENKIAYYDFCNLTQVPPVP 49
ggTLR5    --------------MLHQRLIIVFGIALAG-DICASRSCYSEDQVSMYNSCNLTGVPPVP 45
hsTLR5    ---------------MGDHLDLLLGVVLMAGPVFGIPSCSFDGRIAFYRFCNLTQVPQVL 45
xlTLR5    MDSSAPAAAYNYRIILFYQLTVIIGGSALALDMMTP--CSSINRMANFKFCNLTRMPLVS 58
drTLR5b   --------------MGYTFILILFGLCLNTEVVKSTSVCSVIGYNAICINRGLHQVPELP 46
                               : .::*     :     *   .  :     .*  :* :

              LRR1                   LRR2                    LRR3
acTLR5    EDIVLFTLNFNSIREVKSSSFPLLKELRNLALGTQSVYPVTIRRDAFRNLPNLQKLDLAG 109
ggTLR5    KDTAKLFLTYNYIRQVTATSFPLLEDLFLLEIGTQRVFPLYIGKEAFRNLPNLRVLDLGF 105
hsTLR5    NTTERLLLSFNYIRTVTASSFPFLEQLQLLELGSQYT-PLTIDKEAFRNLPNLRILDLGS 104
xlTLR5    SDTLKLDLSFNYVSEINRTFFPKLYRLVDLNLGSQKTNRLIVKKDSFRNTPNLVKLDLAT 118
drTLR5b   AHVNYVDLSLNSIAELNETSFSRLQDLQFLKVEQQTP-GLVIRNNTFRGLSSLIILKLDY 105
               . *. * :  :. : *. *  *  * :  *    : : .::**. ..*  *.*

                           LRR4                     LRR5
acTLR5    NKMTVLDTGAFLGLLNLRELFLYYNGLNESILEGDYFRDLISLEYLDLQYNKIARLRPHP 169
ggTLR5    NNILLLDLDSFAGLQRLTILRLFQNNLGDSILEERYFQDLRSLEELDLSGNQITKLHPHP 165
hsTLR5    SKIYFLHPDAFQGLFHLFELRLYFCGLSDAVLKDGYFRNLKALTRLDLSKNQIRSLYLHP 164
xlTLR5    NQLLILDPEGLAGLSQLKILFLYYNKLNGSILENDYFKDLTSLEYVDLSSNEIAYLKPNP 178
drTLR5b   NQFLQLETGAFNGLANLELLTLTQCNLDGAVLSGNFFKPLTSLEMLVLRDNNIQKIQPAS 165
          .::  *.  .: ** .*  * *    *. ::*.  :*: * :*  : *  *:*  :   .

                 LRR6                     LRR7
acTLR5    LFFNMNSLGTLKLKLNQIKTICEGDLNSFQGKTFELLSLNSNQLYR---DAVNWTTCGNP 226
ggTLR5    LFYNLTILKAVNLKFNKISNLCESNLTSFQGKHFSFFSLSTNTLYKT--DKMIWAKCPNP 223
hsTLR5    SFGKLNSLKSIDFSSNQIFLVCEHELEPLQGKTLSFFSLAANSLYSR--VSVDWGKCMNP 222
xlTLR5    LFYHLYSLSILILRYNHISSICAGDLHSFEMKNFTFMDLSDNYFYNW--ETLGSDRCGNP 236
drTLR5b   FFLNMRRFHVLDLTFNKVKSICEEDLLNFQGKHFTLLRLSSITLQDMNEYWLGWEKCGNP 225
           * ::  :  : :  *::  :*  :*  :: * : :: *    :      :    * **

               LRR8                     LRR9
acTLR5    FKNIVIKTLDVGSNGWDVATTQQFCAAVQGTPILALELS-HHIMGSSFGFDNLRNPDNDT 285
ggTLR5    FRNITFNSLDVSENGWSTETVQYFCTAIKGTQINYLSFR-SHTMGSGFGFNNLKNPDTDT 282
hsTLR5    FRNMVLEILDVSGNGWTVDITGNFSNAISKSQAFSLILA-HHIMGAGFGFHNIKDPDQNT 281
xlTLR5    FRNIRFDTLLLSGNRFGVSQMQKFSSALNGTKIIQLKLC-HHIMGPGFGYNNFKDPDNRT 295
drTLR5b   FRNSSITTLDLSGNGFKESMAKRFFDAIAGTKIQSLILSNSYNMGSSFGHTNFKDPDNFT 285
          *:*  :  * :. * :       *  *:  :    * :   : **..**. *:::**  *

                 LRR10                    LRR11               LRR12
acTLR5    FVGLAKSGLKLLDLSHGSIFPLSPYVFQSLGDLLWLDLNTNKINQIGKGAFSGLLSLQLL 345
ggTLR5    FTGLARSDLHLLDISNGFIFSLNSLIFESLRNLEFLNLFRNKINQIQKQAFFGLENLEIL 342
hsTLR5    FAGLARSSVRHLDLSHGFVFSLNSRVFETLKDLKVLNLAYNKINKIADEAFYGLDNLQVL 341
xlTLR5    FVGLVNSDLEILDLSKGSIFSMQPYTYGNLTILKVLNLAENKINRIEKDAFYGLNSLINL 355
drTLR5b   FKGLEASGVKTCDLSKSKIFALLKSVFSHFTDLEQLTLAQNEINKIDDDAFWGLTHLLKL 345
          * **  *.:.  *:*:. :*.:    :  :  *  * *  *:**:* . ** **  *  *

                             LRR13                    LRR14
acTLR5    NLSYNLLGEILDYTFVGLHNVISIDLQHNHIGVFGGNPFEYLPKLQEINLRDNALKIIPS 405
ggTLR5    NLSSNLLGELYDYTFEGLHSIMYIDLQQNHIGMIGEKSFSNLVNLKIIDLRDNAIKKLPS 402
hsTLR5    NLSYNLLGELYSSNFYGLPKVAYIDLQKNHIAIIQDQTFKFLEKLQTLDLRDNALTTIHF 401
xlTLR5    NLAHNLLGELYDYSFNSLTVVTVIDLEQNHIGAIQINTFKSLSELNTLNLRGNSMKTITF 415
drTLR5b   NLSQNFLGSIDSRMFENLDKLEVLDLSYNHIRALGDQSFLGLPNLRNLNLTGNAVESVHT 405
          **: *:**.: .  * .*  :  :**. ***  :  :.*  * :*. ::* .*::  :
```

```
                     LRR15                     LRR16                    LRR17
acTLR5   FS---SLLSVFWGGNNRIQSSYNKEVNSA---IVDLEGNRLDDLGYLYKLL-QMPILKYI 458
ggTLR5   FP---HLTSAFLS-DNKLMSVAHTAIVAT---HIELERNWLANLGDLYVLF-QVPGVQYL 454
hsTLR5   IP---SIPDIFLS-GNKLVTLPKINLTAN---LIHLSENRLENLDILYFLL-RVPHLQIL 453
xlTLR5   FESPVSIGYIFVG-GNKLKSIDSSFVYSN---FLDLSENDLRDLGGLYKLL-QYPLLQYV 470
drTLR5b  FAALPNLNKLYLG-KNRISSVSSLPNIAHNLSTLDLEFNKLHALSDLYTILREFPQIENI 464
         :     :   : .  *:: :       :     :.*. * *  *. ** :: . * :: :
                                   LRR18                      LRR19
acTLR5   FLKNNRFSNCQKLNDVPENNQLIYLDLGENMLKLIWERSECLDIFKELSKLQVLHLNNNY 518
ggTLR5   LLKQNRFSYCVKHVDAIENNQLIYMDLGENMLQLVWERGLCLDVFRTLSKLQVLHLNNNY 514
hsTLR5   ILNQNRFSSCSGDQTPSENPSLEQLFLGENMLQLAWETELCWDVFEGLSHLQVLYLNHNY 513
xlTLR5   ILKRNRLSVCYPHFNISKNNSLLHLDLSDNMISLIWDNGQCGNIFSNLSLLGVLKLNNNL 530
drTLR5b  FLQGNTFSSCYNQKQIVLSDKLQLLHLGLSSMQLIWSEEKCLNVFADLHQLQQLSLTANG 524
         :*: * :* *         . .*  : *. . :.* *.   * ::*  *  *  * *. *
                           LRR20                  LRR21
acTLR5   LNFLPEGIFSGLVSLNRLNLDSNLLTYISHNAFPKSLRTLHLSSNQLLYPDPQIFATLDY 578
ggTLR5   LSALPQEIFNGLTSLKRLNLASNLLSHLSLRVFPQSLINLNLSGNQLFSPKPEVFMTLSI 574
hsTLR5   LNSLPPGVFSHLTALRGLSLNSNRLTVLSHNDLPANLEILDISRNQLLAPNPDVFVSLSV 573
xlTLR5   LRYLPNGIFNGLDSLQTLNLSSNLLTYLIPGIFPTNLDTVDLSKNQLYSPNPKLFLSVKT 590
drTLR5b  LQSLPKDIFKDLTSLFFLDLSFNSLKYLPTDVFPKSLQILNLDYNSIYSVDPNLFSTLGY 584
         *  **  :*. * :*  *.*  * *. :    :* .*  :.:. *.:   .*.:* ::
         LRR22                          LRRCT
acTLR5   LDITYNRFYCDCLLSDLVIWLNETNVTLAGSPNDMFCFGPPELATVPLH--QLLVGGCDE 636
ggTLR5   LDITHNKYVCDCALKSLLVWLNETNVTLAGSESDRYCVYPPALAGVPVS--FLTYDDCDE 632
hsTLR5   LDITHNKFICECELSTFINWLNHTNVTIAGPPADIYCVYPDSFSGVSLF--SLSTEGCDE 631
xlTLR5   LDLTDNHYICDCDLVYFLRWLNETNATLLGSPNDIYCMYPTNLLYKPLH--VLEEGDCDE 648
drTLR5b  LSLMNNDFRCDCDLKDFQTWLNQTNVTFVHSIEDVTCASPEDQYMVPVVRSSIQCEDEEE 644
         *.:   * : *:* *   :  ***.**.*:  .   *   *   *       .:    :   . :*
                           TM
acTLR5   DKILEPLQLSLFISTSVVLTMYLAAVVVFTRFRGTCFVWYKTIARTFMKELQSDLDKKKY 696
ggTLR5   DELQQTLRFSVFVFLSVTLLMFLMSTIIFTRCRGICFVWYKTITKTLIGSHPPAADTSEY 692
hsTLR5   EEVLKSLKFSLFIVCTVTLTLFLMTILTVTKFRGFCFICYKTAQRLVFKDHPQGTEPDMY 691
xlTLR5   SEALTTLMFSLFVLNATIILIGMSTVVTYTHYRGFCFVMYKRIISFIIDTEKQEEAADTC 708
drTLR5b  ERRTEKLRLVLFISCTVLIILFTASTIVYISRRGVIFKMYKKLIGELVDEKREEPDPDRF 704
         ..     * : :*:  :. : :   : :     **  *  **      ..          .
                                              TIR            ▼
acTLR5   KYDAYICYSSKDFEWVQNSLIKHLDSQYSDKNRFALCFEDRDFLPGEDHISNIRDAIWNC 756
ggTLR5   MYDAYLCYSKNDFEWVQNSLLKHLDSQYFDKNRFTLCFEERDFLPGEEHINNIRDAIWKS 752
hsTLR5   KYDAYLCFSSKDFTWVQNALLKHLDTQYSDQNRFNLCFEERDFVPGENRIANIQDAIWNS 751
xlTLR5   KYDAYLCYSGKDFQWVQDAFLQNLDTQYSDRNRFHFCFEERDFVPGEDHIVNIRDAIWNS 768
drTLR5b  LYDVYLCFSSKDMKWVERALLKRLDSQFSEHNTLRCCFEERDFIPGEDHLTNMRSAIQNS 764
          **.*:*:* :*: **: :::.**:*: ::* :  ***:***:***::: *::.** :.
                                              TIR              ▼ ▼▼▼▼▼▼▼
acTLR5   RKTICVVTKQFLKDGWCVEAFNFAQSRYFCDLKDVLIMVVAGSLSQYQLMKYQPVRAFLQ 816
ggTLR5   RKTICVVTRQFLKDGWCVEAFNFAQSRYFSDLKEVLIMVVVGSLSQYQLMKHKPIRIFLQ 812
hsTLR5   RKIVCLVSRHFLRDGWCLEAFSYAQGRCLSDLNSALIMVVVGSLSQYQLMKHQSIRGFVQ 811
xlTLR5   KKTICVVTKQFLKDGWCVEALNYAQSRYFTDLKDVLIMVVVGSLSQYQLMKYQPIRAYVK 828
drTLR5b  RKTICVVSEHFLKDGWCLETFTLAQKRMQAELEDILVVLVVGNIPQYRLLKYKQVRSFIE 824
         :* :*:*:.:**:****:*::. ** *   :*:. *:::*.*.:.**:*:*:*:: :* :::
                   TIR
acTLR5   RDRYLRWPEEDQDVEWFLNALSHQILKEKRTQKKAQKKKVPKKTGTLELKVVTIS-- 871
ggTLR5   RSRYLRWPEDYQDIGWFLDNLSSQILKEKKVQRNVS--------GIELQTIATVSH- 860
hsTLR5   KQQYLRWPEDLQDVGWFLHKLSQQILKKEKEKKKDN--------NIPLQTVATIS-- 858
xlTLR5   RCQYLKWPEDIQDVEWFLGRLSYQILKENKVEKKLKK-----SSNHELQTIETIS-- 878
drTLR5b  NRSYLVWPDDGQDLEWFYDQLLHKIRKDIKINQTTKET------KREEANFNTNTAV 875
         .  ** **:: **: **   *  :* *. : ::. .             .. * :
```

Supplementary figure S2. Multiple sequence alignment of vertebrate TLR5 proteins.

Alignment of TLR5 from *Anolis carolinensis* (ac), *Gallus gallus* (gg, chicken, NCBI reference sequence: ABW07794.1), *Homo sapiens* (hs, human, NP003259.2), *Xenopus laevis* (xl, African clawed frog, NP001088449.1) and *Danio rerio* (dr, zebrafish, NP001124067.1). The amino acid sequences were aligned using the ClustalW server with default settings. Asterisks (*) indicate identical residues in all sequences, double dots (:) indicate highly similar residues, single dots (.) indicate similar residues and bars (-) indicate gaps to complete the sequence alignment. acTLR5 leucine rich repeats (LRR 1 to 22) are shaded in gray while acTLR5 signal peptide, N-terminal LRR (LRRNT), C-terminal LRR (LRRCT), transmembrane domain (TM) and TIR domain are shaded in black. ▼ above residues in the TIR domain indicate conserved residues important for TLR5 signalling. Residues in bold and underlined in the drTLR5b sequence are the residues involved in flagellin binding by drTLR5b (adapted from Yoon et al.[1]).

1. Yoon, S. I. *et al.* Structural basis of TLR5-flagellin recognition and signaling. *Science* **335,** 859–64 (2012).
